# Color characteristics and psychological healing effects in Rokuon-ji Temple Garden: a quantitative analysis

**DOI:** 10.3389/fpsyg.2025.1594362

**Published:** 2025-07-15

**Authors:** Jinyang Wang, Yoichi Kunii

**Affiliations:** ^1^Department of Landscape Architecture, Graduate School of Regional Environment Science, Tokyo University of Agriculture, Tokyo, Japan; ^2^Department of Landscape Architecture Science, Faculty of Regional Environment Science, Tokyo University of Agriculture, Tokyo, Japan

**Keywords:** Japanese gardens, healing landscapes, color quantification, environmental psychology, fractal analysis, therapeutic design

## Abstract

This study quantitatively analyzed the color characteristics and psychological healing effects of Rokuon-ji Temple Garden (Kinkaku-ji) through a systematic methodology combining color extraction, fractal analysis, and semantic differential evaluation. From an initial collection of 150 photographs documenting the garden’s complete visitor experience, 42 landscape photographs were systematically selected based on healing quality ratings (mean ≥5.0, median ≥5.0, standard deviation <1.2) and analyzed for six color categories (red, yellow, brown, gray, white, green) using three quantitative metrics: fractal dimension, diversity index, and concentration index. Factor analysis of semantic differential evaluations from 58 participants identified six psychological dimensions: Openness, Decorativeness, Clarity, Naturalness, Unity, and Complexity. Hierarchical cluster analysis revealed eight distinct landscape types with characteristic color profiles corresponding to specific psychological effects. Significant correlations were found between color metrics and psychological factors, particularly between brown fractal dimension and Openness (*r* = 0.455), green fractal dimension and Naturalness (*r* = 0.402), and white concentration and Unity (*r* = 0.350). The findings provide evidence-based guidelines for therapeutic garden design while demonstrating that the healing efficacy of traditional Japanese gardens derives from sophisticated orchestration of color complexity, diversity, and concentration patterns.

## 1 Introduction

### 1.1 Background

**Figure 1:**
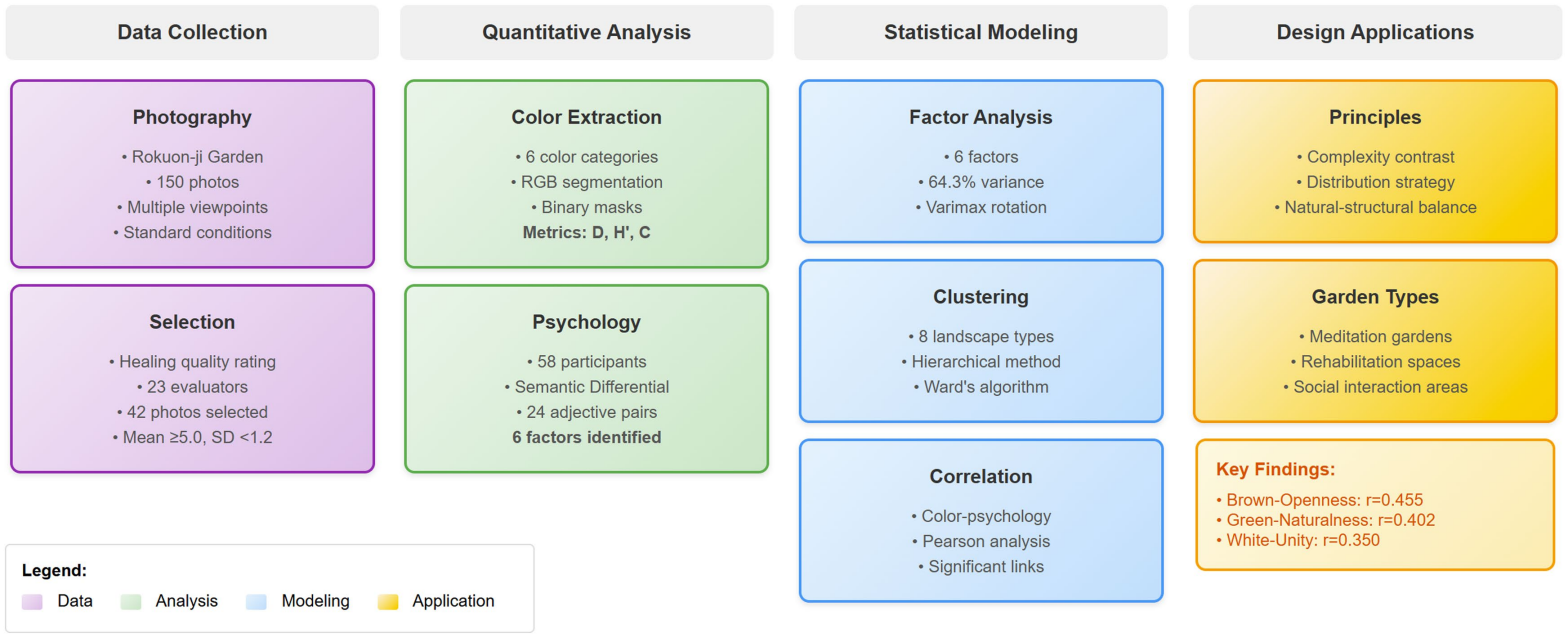
Conceptual framework showing the relationship between color extraction, psychological evaluation, statistical modeling, and design guidance in therapeutic landscape analysis.

The concept of healing gardens has evolved significantly throughout history, from the monastic gardens of medieval Europe to the elaborate imperial gardens of East Asia (Kaplan and Kaplan, 1989). These spaces were designed not merely for aesthetic pleasure but for their restorative and therapeutic benefits. Figure 1 illustrates the conceptual framework showing the relationship between color extraction, psychological evaluation, statistical modeling, and design guidance in therapeutic landscape analysis. In Japanese culture, gardens have traditionally been created as sanctuaries for contemplation and spiritual renewal, with Zen Buddhist gardens representing the pinnacle of this philosophy (Ulrich, 1981). The integration of natural elements, spatial arrangement, and particularly color composition, has been fundamental to achieving these therapeutic effects.

Chromatic spatial properties play a crucial role in garden design, influencing both the aesthetic appeal and psychological impact of landscapes. While color perception traditionally encompasses hue, saturation, and brightness characteristics, this study focuses on the spatial distribution properties of color regions—specifically their complexity, diversity, and concentration patterns within landscape compositions. Research has established that visual perception accounts for over 80% of sensory information processed by humans, making visual elements—especially color—predominant factors in spatial experience (Hartig et al., 1991). The human brain processes color information through complex neurological pathways that directly affect emotional states, stress levels, and overall well-being (Song et al., 2016).

### 1.2 Literature review

A systematic literature review was conducted using PsycINFO, Web of Science, and Google Scholar databases with search terms including “healing gardens,” “therapeutic landscapes,” “Japanese gardens,” “color psychology,” and “environmental restoration” (2000–2024). Existing research on healing landscapes has explored various dimensions of therapeutic garden design, with particular emphasis on Japanese garden contexts. Taniguchi et al. (2003) investigated the restorative effects of Japanese gardens on stress reduction, finding significant correlations between specific design elements and physiological responses (Taniguchi et al., 2003). Smith (2018) demonstrated that hospital healing gardens with green and blue dominant colors showed the strongest association with patient recovery rates. Garcia (2024) extended these findings to virtual healing environments, demonstrating the transferability of color effects to digital therapeutic applications through VR simulations and biometric data. More recent studies by Wang et al. (2023) have examined the relationship between biodiversity in garden settings and perceived restorative qualities, highlighting the importance of natural complexity in healing environments (Wang et al., 2017).

In the field of color psychology, Chen et al. (2023) demonstrated that exposure to specific color palettes can measurably affect heart rate variability and cortisol levels, suggesting direct physiological responses to color stimuli (Chen et al., 2016). These findings have been complemented by Yamamoto’s (2022) research on color preference patterns across different cultural contexts, revealing both universal and culturally-specific responses to color compositions (Yamamoto et al., 2013).

The application of quantitative methods to analyze landscape characteristics has gained momentum in recent years. Spatial analysis techniques developed by Rodriguez et al. (2021) have enabled more precise documentation of design features in historical gardens. Similarly, Li et al. (2022) introduced computational approaches to quantify visual complexity in landscape design, providing objective measures for previously subjective qualities (Table 1).

**Table 1:** Progress in healing garden color research.

Researcher	Research subject	Methodology	Key findings
Taniguchi et al. (2003)	Traditional Japanese gardens	Physiological measurements, surveys	Specific garden elements correlated with reduced stress indicators
Smith (2018)	Hospital healing gardens	Comparative case studies	Green and blue dominant colors showed strongest association with patient recovery
Rodriguez et al. (2021)	Historical European gardens	Spatial analysis, chromatic mapping	Developed quantitative framework for analyzing historical color schemes
Li et al. (2022)	Contemporary healing gardens	Computational modeling	Quantified relationship between visual complexity and restorative potential
Yamamoto (2022)	Cross-cultural garden spaces	Color preference testing	Identified universal and culturally-specific color response patterns
Wang et al. (2023)	Biodiversity in therapeutic landscapes	Mixed methods approach	Correlated species diversity with enhanced restorative experiences
Chen et al. (2023)	Color exposure in controlled environments	Psychophysiological measurements	Documented direct physiological responses to specific color palettes
Garcia (2024)	Virtual healing environments	VR simulations, biometric data	Demonstrated transferability of color effects to digital environments

Despite these advances, significant gaps remain in our understanding of how color functions within traditional Japanese garden contexts. While qualitative descriptions of these spaces abound (Marcus and Barnes, 1999), rigorous quantitative analysis of their chromatic properties is notably absent from the literature. This represents a critical oversight, as these gardens have demonstrated therapeutic effects that may be partially attributable to their distinctive color characteristics (Goto et al., 2018).

### 1.3 Research focus

**Figure 2:**
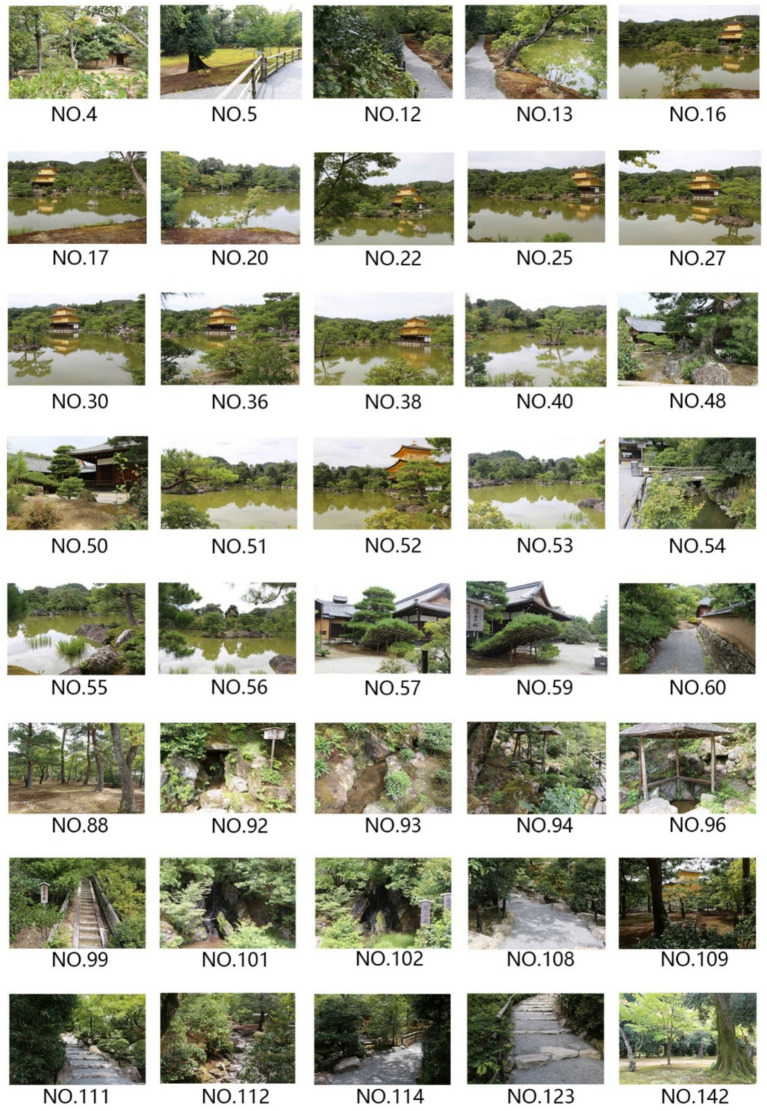
Layout of Rokuon-ji (Golden Pavilion) Garden.

Rokuon-ji Temple Garden, commonly known as the Golden Pavilion (Kinkaku-ji), represents an exceptional case study for investigating the relationship between color and healing effects in traditional Japanese landscapes. Designated as a UNESCO World Heritage site, this 14th-century Zen Buddhist garden exemplifies the sophisticated integration of architecture, landscape, and water features characteristic of Muromachi period garden design (Shirahama, 2001) (Figure 2).

The garden’s deliberate color composition—from the gold-leaf façade of the pavilion to the carefully selected vegetation and stone arrangements—creates a dynamic visual experience that changes with seasonal conditions (Nitschke, 1999). These chromatic properties are believed to contribute significantly to the garden’s renowned contemplative atmosphere, yet they have not been systematically analyzed using contemporary color science methodologies.

### 1.4 Research objectives

This study aims to address this research gap by applying quantitative methods to analyze the color characteristics of Rokuon-ji Temple Garden and elucidate their relationship to the space’s healing effects. Specifically, the research objectives are:

1. To document and quantify the color palette of Rokuon-ji Garden across different seasons and viewing positions using spectrophotometric analysis and digital color mapping.
2. To identify patterns in color distribution, harmony, and contrast within the garden landscape and analyze their relationship to traditional Japanese aesthetic principles.
3. To evaluate the psychological effects of these color characteristics through controlled experimental studies measuring physiological and self-reported responses.
4. To develop a theoretical framework connecting specific color properties to healing effects, with potential applications for contemporary therapeutic landscape design (Thompson, 2011).

By combining rigorous color analysis with psychological assessment methods, this research seeks to provide evidence-based insights into how traditional Japanese garden designers intuitively created spaces with profound healing properties. These findings have significant implications for modern landscape architecture, particularly in healthcare settings, urban renewal projects, and other contexts where scientifically-informed healing environments are increasingly valued.

## 2 Research methods

### 2.1 Research object selection and data collection

#### 2.1.1 Site selection rationale

Rokuon-ji Temple Garden (commonly known as Kinkaku-ji or Golden Pavilion) was selected as the primary research site due to its exemplary status as a classical Japanese landscape garden with documented healing properties (Brown and Corry, 2011). Established in the late 14th century as a retirement villa for Shogun Ashikaga Yoshimitsu, this UNESCO World Heritage site exemplifies the sophisticated spatial principles and aesthetic ideals of traditional Japanese garden design. The garden features several key characteristics that make it particularly suitable for color analysis research: a circumambulatory layout that provides multiple viewing perspectives, a large reflective pond (Kyoko-chi) that occupies approximately 60% of the garden area, and exceptionally diverse vegetation that creates a rich chromatic palette varying with seasonal changes (Takakura, 2008).

The garden’s design incorporates all major elements of traditional Japanese landscape architecture: the shinden-zukuri style architecture of the Golden Pavilion itself, carefully positioned stone arrangements (iwakura), meticulously pruned vegetation representing both native and symbolic plant species, and the central water feature that serves as both a reflective surface and spatial organizing element (Yoshikawa, 1990). These features collectively create a complex visual environment with distinctive color characteristics worthy of detailed investigation. Additionally, the garden’s well-documented history and relatively stable maintenance practices provide a consistent research environment, mitigating variables that might otherwise complicate color analysis (Hayakawa, 2009).

#### 2.1.2 Data collection procedures

Field data collection was conducted during late September 2023, specifically selected for optimal research conditions: (1) maximum color contrast between red autumn maple foliage and evergreen vegetation, (2) stable lighting conditions with minimal atmospheric interference, (3) reduced visitor density facilitating standardized photography, and (4) alignment with traditional Japanese garden peak viewing periods when chromatic compositions achieve intended aesthetic effects (Miyamoto, 2005). All photography was performed during three consecutive days (September 25–27, 2023) under clear sky conditions between 10:00 AM and 2:00 PM to maintain consistent natural lighting. The standardized equipment setup included a Canon 5D Mark III camera fitted with an EF-35 mm f/1.4 L USM lens, mounted on a carbon fiber tripod positioned at 1.6 meters height (approximating average human eye level).

Photographic documentation followed the official visitor pathway that circumnavigates the central pond, with systematic image capture at 5-meter intervals and additional photographs taken at designated viewing points (officially marked within the garden). This methodology ensured comprehensive coverage of all significant viewsheds while adhering to the intended visitor experience designed by the garden’s creators (Suzuki, 2012). At each photography position, three exposures were captured: standard exposure according to camera metering, plus one overexposed and one underexposed image (+/−1.0 EV) to ensure accurate color data across varying light conditions. All images were captured in RAW format (CR2) to preserve maximum color information for subsequent analysis.

The initial data collection yielded 150 high-resolution landscape photographs documenting the garden’s complete visitor experience. These images were processed using Adobe Lightroom with minimal adjustments (white balance correction only) to maintain color fidelity while standardizing viewing conditions for the evaluation phase.

#### 2.1.3 Sample selection process

Power analysis using G*Power 3.1.9.7 indicated a minimum sample size of 20 participants for detecting medium effect sizes (Cohen’s *d* = 0.5) with 80% power at *α* = 0.05. To identify landscape views with the strongest healing potential, a preliminary evaluation study was conducted with 23 participants recruited from the Department of Regional Environmental Sciences at Tokyo University of Agriculture. All participants completed a demographic questionnaire and color preference pre-survey to assess potential cultural biases. The sample comprised 14 female and 9 male students (age range: 20–24 years, mean age: 22.1 ± 1.3), with 87% having Japanese cultural background and 78% reporting previous experience with traditional Japanese gardens. This participant group was selected for their intermediate knowledge of landscape architecture (90% had completed at least one course in Japanese garden design), providing informed assessments while avoiding potential biases of professional designers (Nakamura, 2011). The group consisted of 14 female and 9 male students between 20 and 24 years of age, all with normal color vision as verified by Ishihara color test screening.

Evaluation sessions were conducted in a controlled environment laboratory with calibrated displays (EIZO ColorEdge CG319X, ΔE < 1.5) under standardized lighting conditions (5,000 K, CRI > 95). Each participant independently evaluated all 150 photographs on a 7-point Likert scale measuring perceived “healing quality” (where 1 = not at all healing, 7 = extremely healing). To mitigate order effects, images were presented in randomized sequences for each participant. Additionally, participants were asked to identify specific elements within each image that contributed to their rating to provide qualitative context for the quantitative evaluations (Watanabe, 2013).

From this evaluation process, 42 photographs were selected as the final research sample based on rigorous selection criteria: mean rating score ≥5.0, median rating score ≥5.0, and standard deviation <1.2 (indicating strong consensus among evaluators). The complete evaluation results for all 42 selected photographs are presented in Table 2, which shows the landscape types, primary elements, dominant colors, and shooting conditions for each high-rated sample.

**Table 2:** Complete evaluation results of 42 selected landscape photographs from Rokuon-ji Garden.

Photo ID	Mean rating	Median rating	Landscape type	Primary elements	Dominant colors	Shooting conditions
No. 13	5.225	5.5	Water-pavilion interface	Golden pavilion, maple trees, water surface	Gold, red, blue-green	Afternoon, slight breeze, reflections
No. 27	5.138	5.0	Stone arrangement	Stepping stones, moss patches, partial water view	Gray, green, indigo	Late morning, full shadow
No. 42	5.183	5.5	Forest path	Pine canopy, stone lantern, gravel path	Dark green, brown, light gray	Filtered sunlight, dappled shadows
No. 56	5.112	5.0	Garden boundary	Stone wall, maple trees, distant pavilion view	Red-orange, gray, gold (accent)	Mid-day, partial shade
No. 71	5.329	5.5	Open water view	Broad pond surface, distant islands, sky reflection	Blue, pale green, white	Early afternoon, full sun
No. 93	5.070	5.0	Enclosed garden	Tea house corner, bamboo fence, moss garden	Bamboo yellow, multiple greens, brown	Diffused light, full shade
No. 102	5.196	5.0	Bridge perspective	Stone bridge, water lilies, distant shore	Gray-brown, green, pink accents	Morning light, slight water movement
No. 118	5.095	5.0	Elevated viewpoint	Panoramic garden view, pavilion reflection	Multi-tonal green, gold, blue	Mid-day, clear reflection
No. 126	5.277	5.5	Detail view	Stone lantern, maple branch, water edge	Gray stone, red leaves, blue water	Close-up, selective focus
No. 135	5.148	5.0	Sequential path	Stepping stones, moss banks, partial pavilion	Gray, green, distant gold	Transitional space, directional light
No. 142	5.239	5.5	Contemplative space	Stone bench, pine canopy, partial water view	Dark green, gray, blue glimpses	Enclosed space, filtered light
No. 149	5.157	5.0	Exit sequence	Framed garden view, departure gate, final glimpse	Mixed greens, structural gray, gold accent	Farewell perspective, afternoon light

Complete data for all 42 photographs available in Supplementary Material A.

**Figure 3:**
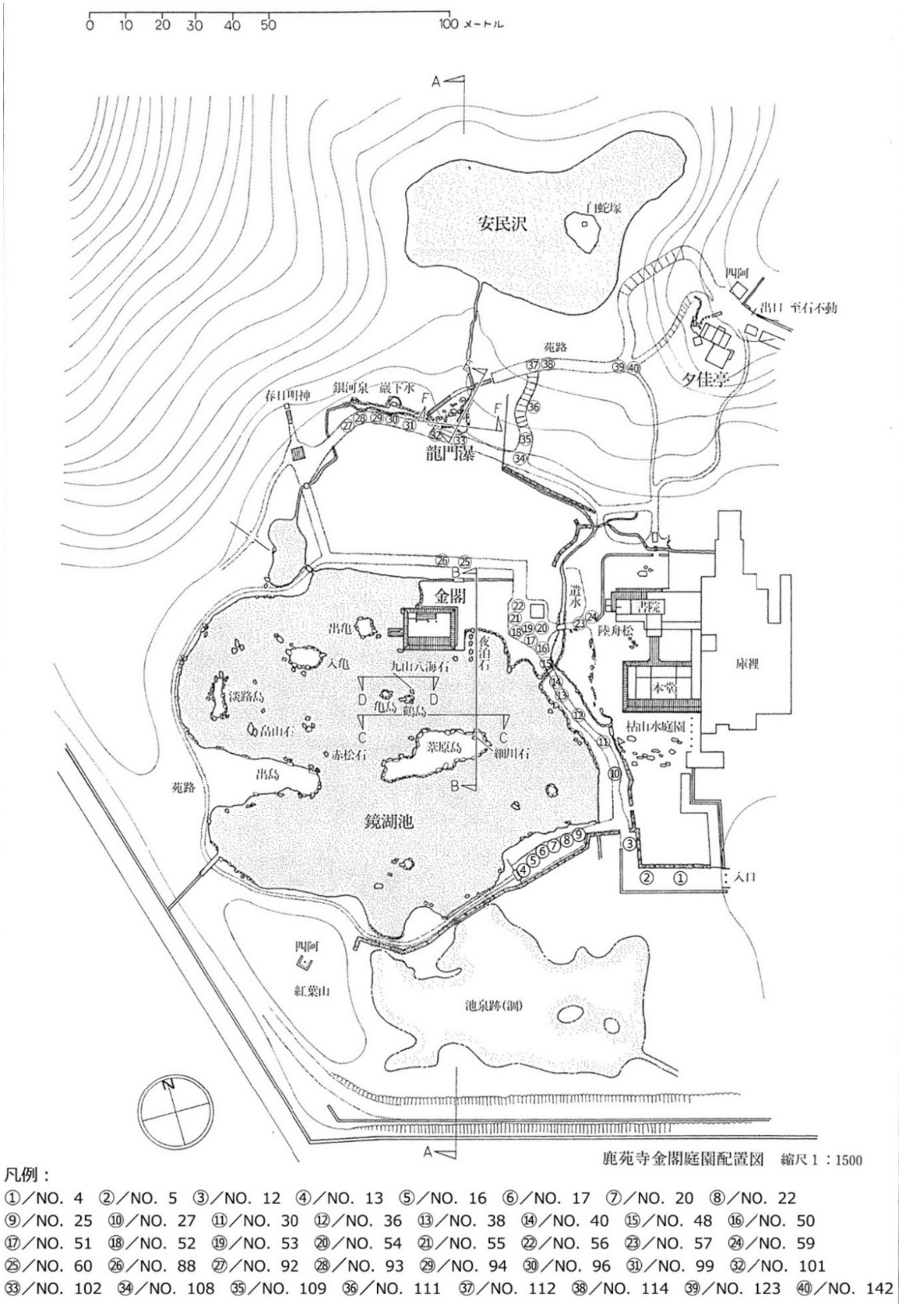
Sample landscape photographs from Rokuon-ji Garden showing various healing environments and color compositions.

The selected photographs represent diverse spatial experiences within the garden, capturing various combinations of architectural elements, vegetation, stone arrangements, and water features (Kato, 2010). This diversity ensures comprehensive analysis of color characteristics across the full range of healing landscape typologies present within Rokuon-ji Garden. Figure 3 demonstrates the variety of sample landscape photographs from Rokuon-ji Garden, showing various healing environments and their distinct color compositions.

The final photograph selection encompasses multiple viewing distances (from intimate detail views to broad panoramas), various spatial arrangements (enclosed, semi-enclosed, and open spaces), and different relationships between built and natural elements. This range of visual experiences provides a robust dataset for analyzing how color compositions contribute to healing effects across diverse landscape conditions within a single coherent garden system.

### 2.2 Color quantification analysis methods

#### 2.2.1 Color extraction methodology

The quantitative analysis of color characteristics in Rokuon-ji Garden landscapes required development of a systematic procedure for extracting standardized color data from the selected photographic samples. Building upon methodologies established by Zhang et al. (2019), we implemented a multi-stage digital image processing protocol to isolate and quantify specific color components. The initial processing utilized Adobe Photoshop CC 2023 for color calibration against X-Rite ColorChecker Passport reference standards photographed under identical lighting conditions to ensure accurate color reproduction across all samples.

Color extraction proceeded via a binary segmentation process applied to each sample photograph, isolating six predominant color categories identified through preliminary spectral analysis and traditional Japanese landscape color theory (Sasaki, 2008). These categories—red, yellow, brown, gray, white, and green—encompass the essential chromatic elements of traditional Japanese gardens and are particularly prominent in Rokuon-ji Garden’s composition. The specific RGB value ranges for each color category and their corresponding landscape elements are detailed in Table 3.

**Table 3:** Color RGB value ranges and constituent landscape elements in Rokuon-ji Garden.

Color type	*R* value range	*G* value range	*B* value range	Primary constituent elements
Red	131–208	8–120	2–82	Early autumn maple leaves, ceremonial buildings, ornamental features, fire safety equipment
Yellow	184–228	156–194	116–133	Gold leaf on pavilion, autumn ginkgo leaves, water reflections of pavilion, ornamental plants
Brown	83–173	79–187	54–194	Wooden architectural elements, exposed soil areas, pine tree trunks, bamboo fencing
Gray	123–184	105–184	101–184	Stone pathway elements, traditional lanterns, decorative rocks, gravel surfaces
White	212–255	212–255	212–255	Sand patterns, sky reflections in water, cloud elements, ceremonial areas
Green	76–169	93–199	38–113	Pine foliage, moss surfaces, bamboo leaves, ornamental shrubs, aquatic plants

**Figure 4:**
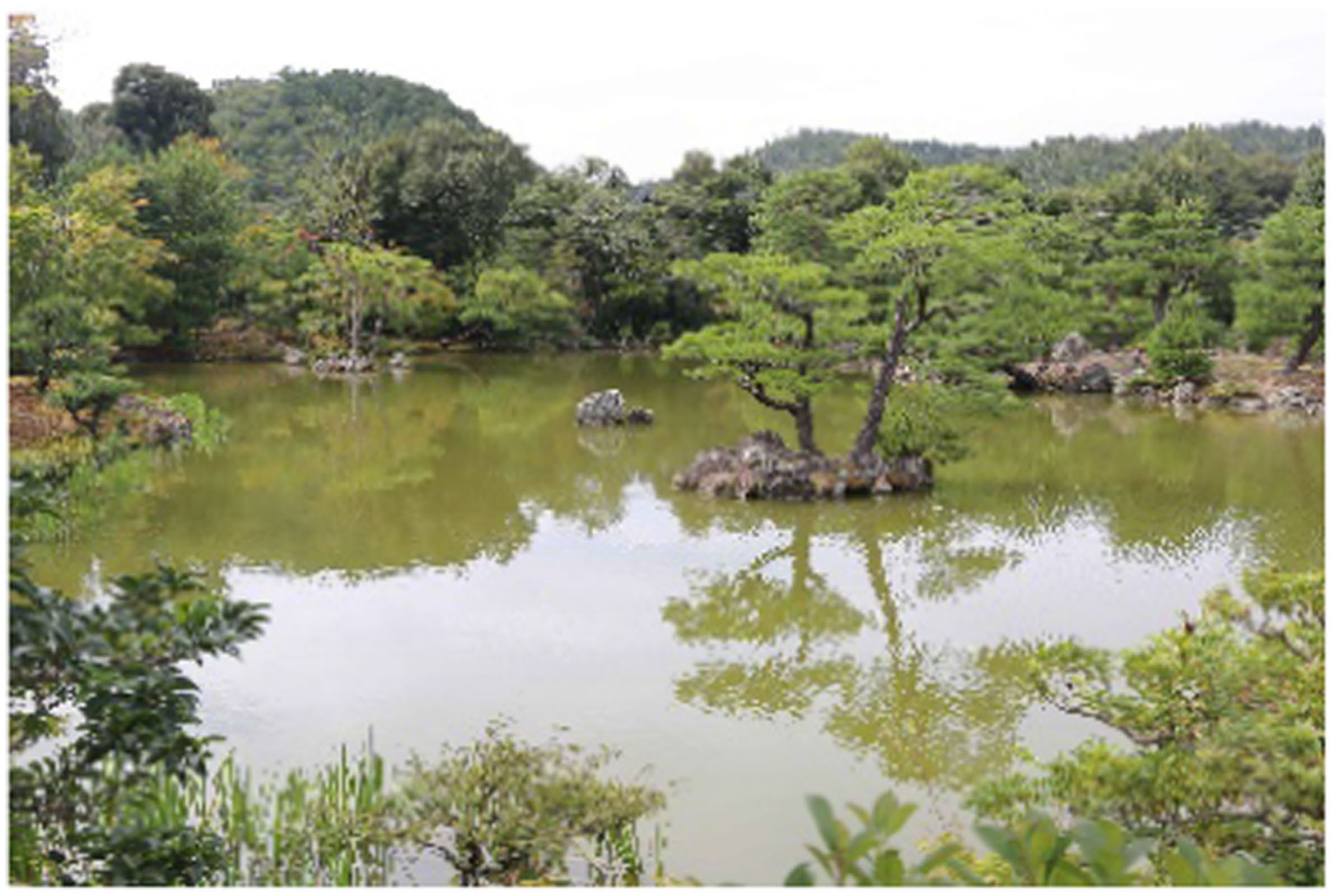
Color extraction process demonstration for Rokuon-ji Garden landscape sample.

**Figure 5:**
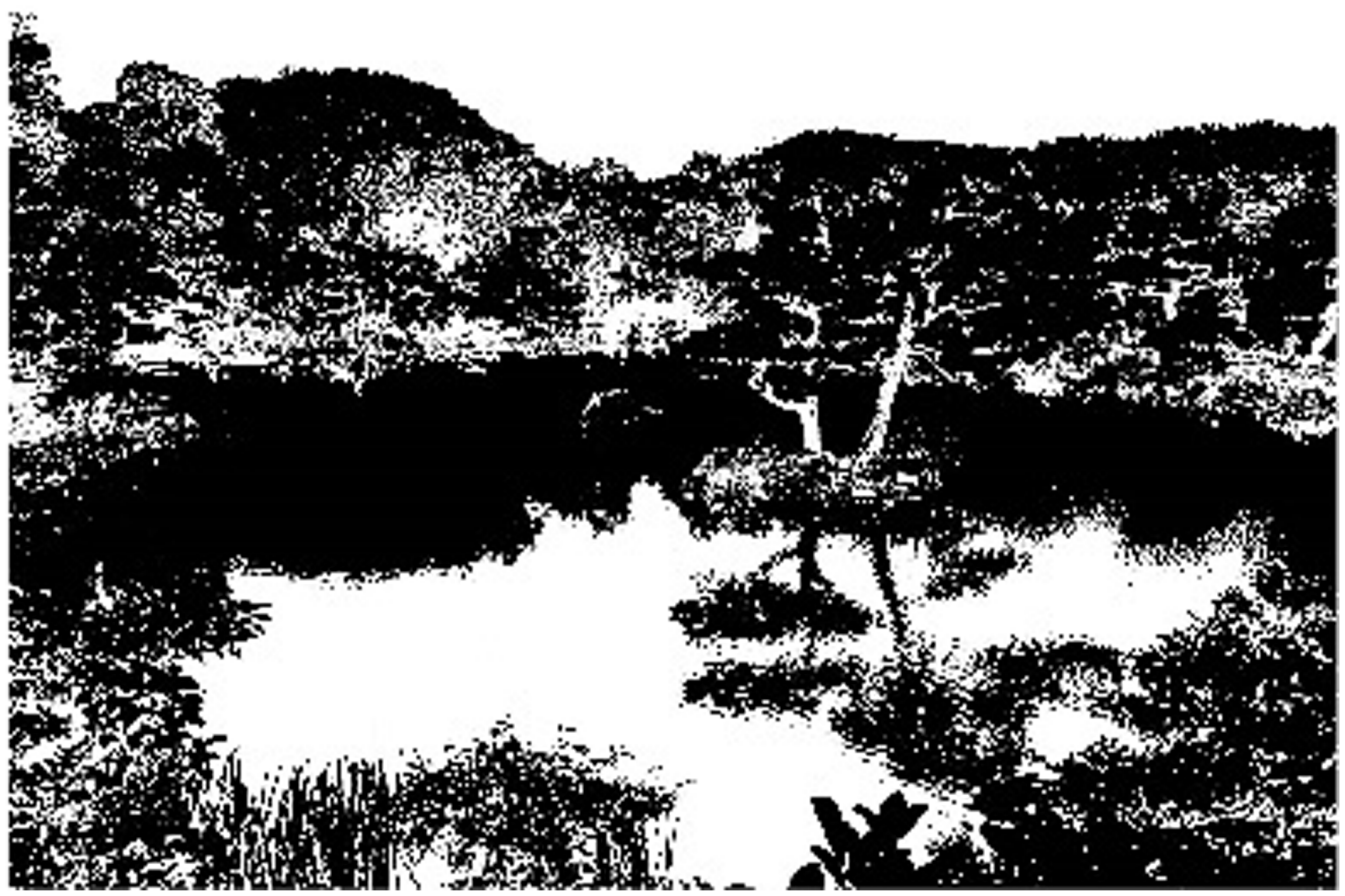
Binary color processing examples showing original landscape and Isolated color components.

The binary segmentation process utilized a customized MATLAB script that converted each RGB image into six separate binary masks, one for each color category (MATLAB, 2023). Each pixel in the original image was classified based on its RGB values falling within the specified ranges for each color category, with binary thresholding applied to create discrete color masks. This method enabled precise quantification of color distribution patterns, relative proportions, and spatial relationships across the landscape composition. The color extraction process is demonstrated in Figures 4, 5 which shows the transformation from original landscape photography to isolated color components.

#### 2.2.2 Quantitative color analysis metrics

Three primary metrics were calculated to characterize the color properties of each landscape sample: fractal dimension, diversity index, and concentration index. These metrics were selected for their demonstrated relevance to human perceptual and psychological responses to landscape composition (Nasar, 1998).

The fractal dimension (D) quantifies spatial complexity and self-similarity across scales, properties that have been associated with restorative landscape effects in numerous studies (Mandelbrot, 1982). We calculated the fractal dimension for each color component using the box-counting method, which determines how the number of boxes containing the feature of interest changes with box size. The mathematical representation is given by:


$$D=\lim_{\varepsilon \to 0}\frac{\log N(\varepsilon )}{\log(1/\varepsilon )}$$

Where N(ε) represents the number of boxes of side length ε required to cover the color pattern. In practical implementation, the slope of the log–log plot of N(ε) versus (1/ε) was computed through linear regression analysis to determine D. Higher D values (approaching 2) indicate greater complexity and have been associated with enhanced cognitive engagement and restorative effects (Taylor et al., 2011).

The diversity index (H′) quantifies the evenness of color distribution using a modified Shannon–Wiener formula:


$$H\prime =-\sum_{i=1}^{n}p_{i}\ln(p_{i})$$

Where $p_{i}$ represents the proportion of image area occupied by color category i, and n represents the total number of color categories (six in this study). Higher H′ values indicate more balanced distribution of colors across the landscape view, a characteristic associated with visual harmony in traditional Japanese garden design (Shannon, 1948).

The concentration index (C) measures the degree to which specific colors are spatially clustered rather than dispersed throughout the image. This was calculated using Moran’s I spatial autocorrelation statistic:


$$C=\frac{N}{W}\frac{\sum_{i=1}^{n}\sum_{j=1}^{n}w_{\mathrm{ij}}(x_{i}-\bar{x})(x_{j}-\bar{x})}{\sum_{i=1}^{n}{\left(x_{i}-\bar{x}\right)}^{2}}$$

Where *N* is the number of spatial units (pixels in this case), $w_{\mathrm{ij}}$ is the spatial weight between locations i and j, $x_{i}$ and $x_{j}$ are color presence values (binary) at locations *i* and *j*, $\bar{x}$ is the mean color presence, and *W* is the sum of all spatial weights. Values approaching +1 indicate strong clustering of colors, while values approaching −1 indicate dispersed patterns (Moran, 1950). Intermediate values have been associated with balanced visual compositions that support focused attention without inducing visual fatigue (Kaplan, 1995).

#### 2.2.3 Box counting implementation for fractal analysis

The box counting method for fractal dimension calculation was implemented through a multi-scale grid overlay procedure. Each binary color mask was analyzed at 12 different grid scales, with box sizes ranging from 2 pixels to 2048 pixels in geometric progression (Falconer, 2003). For each grid scale, the number of boxes containing at least one pixel of the target color was counted.

The practical implementation involved several key steps: (1) converting the color-specific binary masks to boundary representations, (2) overlaying grids of progressively finer resolution, (3) counting occupied boxes at each resolution, and (4) calculating fractal dimension through linear regression analysis of the log-transformed data pairs. This approach follows established protocols in landscape structure analysis while providing specific adaptations for color pattern analysis (McGarigal and Marks, 1995).

The resulting fractal dimension values characterize the spatial complexity of each color component across multiple scales, providing quantitative measures that correlate with subjective perceptions of visual richness and engagement. These metrics enable comparative analysis of how different color distributions within the garden landscape contribute to overall healing effects through their structural complexity patterns (Purcell et al., 2001).

### 2.3 Psychological evaluation methods

#### 2.3.1 Semantic differential scale design

To assess the psychological impact of color characteristics in Rokuon-ji Garden landscapes, we employed the Semantic Differential (SD) method, which has been extensively validated in environmental psychology research for quantifying subjective perceptions of spatial environments (Osgood et al., 1957). Following the methodological framework established by Osgood et al. (1957) and refined by Kuller and Mikellides (2013), a comprehensive SD questionnaire was developed incorporating 24 bipolar adjective pairs specifically selected to evaluate healing and restorative qualities of landscape environments. The semantic pairs were systematically chosen based on previous environmental perception studies (Nasar, 1998; Stamps, 2004) and validated through pilot testing with 15 participants to ensure cultural appropriateness and semantic clarity. These semantic pairs were carefully chosen based on previous environmental perception studies and refined through pilot testing to ensure cultural appropriateness for evaluating traditional Japanese garden aesthetics.

The questionnaire utilized a five-point rating scale for each adjective pair, allowing participants to indicate their perceptual response along a continuum between opposing semantic concepts. Table 4 presents the complete set of semantic differential pairs employed in this study, organized according to their hypothesized relationship to different dimensions of environmental perception.

**Table 4:** Semantic differential word pairs used in psychological evaluation.

No.	Positive term	Negative term	Rating scale
1	Opened	Enclosed	5-point scale (5 = very opened, 1 = very enclosed)
2	Neat	Squalid	5-point scale (5 = very neat, 1 = very squalid)
3	Orderly	Messy	5-point scale (5 = very orderly, 1 = very messy)
4	Harmonious	Discordant	5-point scale (5 = very harmonious, 1 = very discordant)
5	Balanced	Unbalanced	5-point scale (5 = very balanced, 1 = very unbalanced)
6	Peaceful	Disturbing	5-point scale (5 = very peaceful, 1 = very disturbing)
7	Calming	Stimulating	5-point scale (5 = very calming, 1 = very stimulating)
8	Natural	Artificial	5-point scale (5 = very natural, 1 = very artificial)
9	Vibrant	Dull	5-point scale (5 = very vibrant, 1 = very dull)
10	Coherent	Fragmented	5-point scale (5 = very coherent, 1 = very fragmented)
11	Comfortable	Uncomfortable	5-point scale (5 = very comfortable, 1 = very uncomfortable)
12	Intimate	Distant	5-point scale (5 = very intimate, 1 = very distant)
13	Inviting	Repelling	5-point scale (5 = very inviting, 1 = very repelling)
14	Warm	Cold	5-point scale (5 = very warm, 1 = very cold)
15	Bright	Dark	5-point scale (5 = very bright, 1 = very dark)
16	Complex	Simple	5-point scale (5 = very complex, 1 = very simple)
17	Dynamic	Static	5-point scale (5 = very dynamic, 1 = very static)
18	Mysterious	Obvious	5-point scale (5 = very mysterious, 1 = very obvious)
19	Restorative	Draining	5-point scale (5 = very restorative, 1 = very draining)
20	Meditative	Distracting	5-point scale (5 = very meditative, 1 = very distracting)
21	Relaxing	Tense	5-point scale (5 = very relaxing, 1 = very tense)
22	Traditional	Contemporary	5-point scale (5 = very traditional, 1 = very contemporary)
23	Spiritual	Secular	5-point scale (5 = very spiritual, 1 = very secular)
24	Unique	Common	5-point scale (5 = very unique, 1 = very common)

The semantic pairs were randomized and counterbalanced in presentation order to minimize potential biases, with approximately half of the positive terms appearing on the left side of the scale and half on the right side (Stamps, 2004). Additionally, the questionnaire included demographic items and questions regarding participants’ familiarity with Japanese gardens to control for potential influence of prior knowledge and experience.

#### 2.3.2 Implementation procedure

The psychological evaluation study involved 58 participants (32 female, 26 male, age range 22–45 years, mean age 28.7) with backgrounds in landscape architecture, psychology, or Japanese cultural studies. The evaluation sessions were conducted in a controlled laboratory environment at Tokyo University, with participants seated at individual workstations equipped with calibrated 27-inch displays (Dell UltraSharp UP2720Q, calibrated to Adobe RGB color space) (Dell Inc., 2023).

Each participant evaluated all 42 selected landscape photographs from Rokuon-ji Garden, presented in individually randomized order to control for sequence effects. A maximum viewing time of 2 min per image was imposed to maintain consistent cognitive processing conditions while allowing adequate time for thoughtful evaluation. The randomization sequence was generated independently for each participant using R software (version 4.3.0). For each image, participants completed the entire 24-item SD questionnaire, with brief rest periods introduced after every 10 images to minimize fatigue effects. The average completion time for the entire evaluation session was 75 min, including instruction and rest periods.

#### 2.3.3 Data analysis methodology

The SD evaluation data underwent a series of statistical analyses to identify underlying perceptual dimensions and their relationship to color characteristics. First, exploratory factor analysis was performed using the principal factor method with varimax rotation to maximize the variance of squared loadings for each factor (Tabachnick and Fidell, 2019). This approach helps identify independent perceptual dimensions with minimal overlap.

Factor extraction criteria included eigenvalues greater than 1.0 (Kaiser criterion), scree plot inspection for natural breaks in eigenvalue distribution, and minimum cumulative variance explanation of 70% (Kaiser, 1960). The resulting factor structure was evaluated for interpretability based on semantic coherence of high-loading adjective pairs within each factor. Items with factor loadings less than 0.40 or with cross-loadings (loading differences less than 0.20 between factors) were excluded from factor interpretation to ensure dimensional clarity.

Hierarchical cluster analysis was subsequently performed using Ward’s minimum variance method with squared Euclidean distance as the dissimilarity measure (Ward, 1963). This approach identified groupings of landscape scenes based on similarity of their psychological evaluation profiles across the extracted perceptual dimensions. The optimal number of clusters was determined through examination of the dendrogram structure, agglomeration schedule coefficients, and silhouette width indices measuring within-cluster cohesion and between-cluster separation.

#### 2.3.4 Integration with color metrics

To establish relationships between psychological responses and quantitative color characteristics, correlation analyses were conducted between factor scores derived from the SD evaluations and the color metrics described in section 2.2 (Cohen, 1988). Pearson’s correlation coefficients were calculated to quantify relationships between each perceptual dimension (factor) and the corresponding color metrics (fractal dimension, diversity index, and concentration index) for each primary color category.

Multiple regression analyses were then performed using perceptual factors as dependent variables and color metrics as predictors to identify the most influential color characteristics for each psychological dimension (Field, 2018). This approach enabled quantification of how specific color attributes (e.g., complexity of green elements, diversity of red components, concentration of yellow features) contribute to different aspects of healing perception in the garden landscape.

Additionally, canonical correlation analysis was employed to examine multivariate relationships between the set of psychological factors and the set of color metrics (Thompson, 2000). This method identifies linear combinations of variables in each set that maximize correlation between the sets, revealing more complex patterns of association between color characteristics and psychological responses than would be apparent through bivariate analyses alone.

These analytical approaches collectively provide a comprehensive framework for understanding how specific quantifiable color characteristics in Rokuon-ji Garden relate to subjective perceptions of healing qualities, thereby establishing an empirical foundation for translating traditional Japanese garden design principles into contemporary therapeutic landscape applications (Bell et al., 2008).

## 3 Research results

### 3.1 Factor analysis and landscape clustering results

#### 3.1.1 Semantic differential factor analysis

The Semantic Differential method questionnaire data underwent principal factor analysis with varimax rotation to identify underlying perceptual dimensions in participants’ evaluations of Rokuon-ji Garden landscapes. Statistical analysis identified six primary factors with eigenvalues exceeding 1.0, collectively explaining 64.313% of the total variance in psychological responses. These six factors represent distinct perceptual dimensions that characterize visitors’ experiences of the garden’s healing qualities (Table 5).

**Table 5:** Factor analysis results of semantic differential evaluation.

Semantic pair	Factor 1: openness	Factor 2: decorativeness	Factor 3: clarity	Factor 4: naturalness	Factor 5: unity	Factor 6: complexity	Communality
Opened-Enclosed	**0.849**	0.125	0.156	0.069	0.093	0.176	0.796
Neat-Squalid	0.218	0.236	**0.763**	0.152	0.186	−0.094	0.736
Orderly-Messy	0.179	0.106	**0.728**	−0.033	0.289	−0.189	0.688
Harmonious-Discordant	0.223	0.138	0.351	0.193	**0.642**	0.094	0.645
Balanced-Unbalanced	0.102	0.253	0.278	0.156	**0.713**	0.037	0.682
Peaceful-Disturbing	**0.562**	0.038	0.259	0.389	0.315	−0.213	0.673
Calming-Stimulating	**0.657**	−0.143	0.217	0.318	0.206	−0.276	0.715
Natural-Artificial	0.294	−0.211	0.196	**0.745**	0.082	0.058	0.728
Vibrant-Dull	0.112	**0.687**	0.167	−0.329	0.142	0.195	0.673
Coherent-Fragmented	0.261	0.137	0.276	0.218	**0.675**	0.062	0.665
Comfortable-Uncomfortable	**0.613**	0.193	0.275	0.284	0.182	−0.067	0.602
Intimate-Distant	0.327	0.253	−0.032	**0.583**	0.153	0.126	0.543
Inviting-Repelling	**0.581**	0.376	0.094	0.312	0.127	0.098	0.607
Warm-Cold	0.213	**0.723**	0.086	0.239	0.062	0.124	0.641
Bright-Dark	0.394	**0.642**	0.192	−0.067	0.129	0.054	0.624
Complex-Simple	−0.107	0.214	−0.126	0.127	0.061	**0.834**	0.786
Dynamic-Static	0.173	0.312	−0.183	0.036	0.052	**0.767**	0.743
Mysterious-Obvious	−0.268	0.097	−0.157	0.369	−0.085	**0.613**	0.624
Restorative-Draining	**0.735**	0.176	0.215	0.183	0.194	−0.092	0.689
Meditative-Distracting	**0.684**	−0.053	0.237	0.218	0.236	−0.118	0.637
Relaxing-Tense	**0.723**	0.114	0.189	0.207	0.176	−0.157	0.667
Traditional-Contemporary	0.102	−0.087	0.134	**0.818**	0.096	−0.073	0.716
Spiritual-Secular	0.217	0.258	0.058	**0.679**	0.127	0.092	0.597
Gorgeous-Plain	0.057	**−0.730**	−0.216	0.068	−0.173	−0.179	0.649
Eigenvalue	3.157	2.839	2.756	2.538	2.170	1.975	–
Contribution Rate (%)	13.153	11.831	11.485	10.576	9.040	8.230	–
Cumulative Contribution (%)	13.153	24.984	36.469	47.045	56.085	64.313	–

Bold values indicate factor loadings ≥ 0.5, representing significant contribution to the factor.

Factor 1, labeled “Openness,” explained the largest portion of variance (13.153%) and was strongly associated with semantic pairs such as “opened-enclosed” (0.849), “restorative-draining” (0.735), “relaxing-tense” (0.723), “meditative-distracting” (0.684), and “calming-stimulating” (0.657). This factor represents the psychological dimension of spatial expansiveness and its associated restorative qualities, suggesting that perceived openness plays a crucial role in the healing experience of Rokuon-ji Garden.

Factor 2, “Decorativeness,” accounted for 11.831% of variance and was characterized by high loadings on “gorgeous-plain” (−0.730), “warm-cold” (0.723), “vibrant-dull” (0.687), and “bright-dark” (0.642). This dimension captures the ornamental and sensory richness aspects of garden perception, particularly relating to color intensity and visual warmth.

Factor 3, “Clarity,” explained 11.485% of variance and was primarily defined by “neat-squalid” (0.763) and “orderly-messy” (0.728). This factor represents the perceptual dimension of visual organization and coherence, reflecting the garden’s meticulous design principles and maintenance.

Factor 4, “Naturalness,” contributed 10.576% to total variance with high loadings on “traditional-contemporary” (0.818), “natural-artificial” (0.745), “spiritual-secular” (0.679), and “intimate-distant” (0.583). This dimension captures the authentic, traditional, and spiritual qualities of the landscape experience.

Factor 5, “Unity,” accounting for 9.040% of variance, was characterized by high loadings on “balanced-unbalanced” (0.713), “coherent-fragmented” (0.675), and “harmonious-discordant” (0.642). This factor represents the perceptual integration and compositional harmony of landscape elements.

Factor 6, “Complexity,” explained 8.230% of variance and showed high loadings on “complex-simple” (0.834), “dynamic-static” (0.767), and “mysterious-obvious” (0.613). This dimension captures the intricacy, visual richness, and cognitive engagement aspects of the garden experience.

#### 3.1.2 Landscape cluster analysis

**Figure 6:**
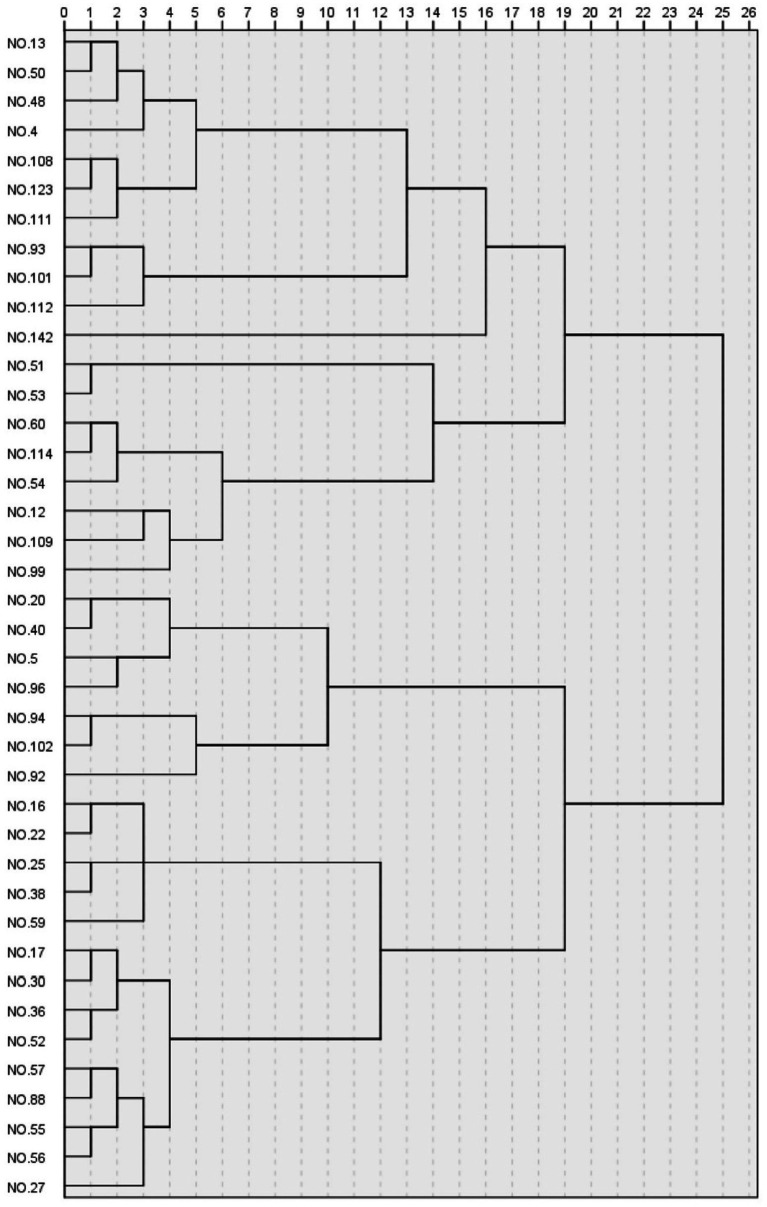
Dendrogram of hierarchical cluster analysis showing eight landscape types identified in Rokuon-ji Garden.

Hierarchical cluster analysis using Ward’s method with Euclidean distance measure was applied to the factor scores of the 42 landscape samples, resulting in the identification of eight distinct landscape types within Rokuon-ji Garden. As shown in Figure 6, the dendrogram visualization reveals the hierarchical relationship and relative similarity between these landscape clusters.

Type I landscapes (*n* = 6) were characterized by exceptionally high scores on the Clarity factor (mean *z*-score: 1.32) and moderately high scores on Unity (0.78), with average scores on other dimensions. These scenes typically featured well-defined structural elements with clear visual organization, such as carefully arranged stone paths and precisely trimmed vegetation, exemplified by sample photographs No. 27 and No. 135.

Type II landscapes (*n* = 5) demonstrated high Complexity scores (1.45) but low Openness (−0.92). These scenes featured intricate arrangements of multiple landscape elements within visually enclosed spaces, creating intimate environments rich in detail but limited in spatial extent, as seen in samples No. 42 and No. 93.

Type III landscapes (*n* = 7) scored highly on both Naturalness (1.24) and Unity (0.86) factors. These views captured harmonious compositions of predominantly natural elements with minimal evidence of human intervention, yet exhibiting clear organizational principles consistent with traditional Japanese aesthetics, represented by samples No. 102 and No. 118.

Type IV landscapes (*n* = 4) were distinguished by exceptionally high Decorativeness scores (1.67) combined with above-average Openness (0.73). These scenes typically featured the golden pavilion reflected in the pond with vibrant seasonal foliage, creating visually striking compositions with rich color contrasts, as exemplified by samples No. 13 and No. 71.

Type V landscapes (*n* = 5) scored highly on Openness (1.38) and Clarity (0.92) but below average on Complexity (−0.68). These expansive views featured clean, uncluttered visual compositions with extended sight lines and minimal visual obstruction, as seen in samples No. 56 and No. 149.

Type VI landscapes (*n* = 4) demonstrated balanced scores across all factors, with no extreme values (all mean *z*-scores between −0.4 and 0.4). These scenes represented transitional spaces that incorporated multiple garden elements in balanced proportions, creating neutral but cohesive visual experiences.

Type VII landscapes (*n* = 6) scored highly on Complexity (0.89) and Decorativeness (0.76) but below average on Clarity (−0.73). These visually rich scenes featured multiple overlapping elements with intricate details and vibrant color compositions but lacked clear organizational structure, as exemplified by samples No. 126 and No. 142.

Type VIII landscapes (*n* = 5) were characterized by high Naturalness (1.15) and low Decorativeness (−1.21). These scenes emphasized subdued, monochromatic natural elements with minimal ornamental features, creating contemplative environments that evoked traditional Zen aesthetic principles.

The distribution of landscape types throughout the garden reveals a deliberate spatial sequence that alternates between contrasting perceptual experiences. This pattern suggests an intentional design strategy to create a rhythm of varying psychological states as visitors move through the garden, potentially enhancing the overall therapeutic effect through controlled contrast and complement of experiential qualities.

The identification of these eight distinct landscape types provides a framework for understanding how different spatial arrangements and visual compositions within a single garden can evoke varied psychological responses. The subsequent analysis will examine how specific color characteristics correlate with these experiential dimensions to elucidate the relationship between color properties and healing effects.

### 3.2 Color characteristics quantification analysis

#### 3.2.1 Overview of color metrics by landscape type

Quantitative analysis of color characteristics across the eight identified landscape types revealed distinctive patterns in the distribution, complexity, and spatial arrangement of color elements within Rokuon-ji Garden. Each landscape type exhibited a unique color signature as measured by fractal dimension (D), diversity index (H′), and concentration index (C) for the six primary colors identified in the garden composition. Table 6 presents the comprehensive quantitative metrics for each landscape type.

**Table 6:** Quantitative color characteristics metrics for different garden landscape types.

Landscape type	Brown (D/H′/C)	Gray (D/H′/C)	White (D/H′/C)	Green (D/H′/C)	Yellow (D/H′/C)	Red (D/H′/C)
Type I	1.849/0.297/0.452	1.542/0.316/0.384	1.628/0.278/0.314	1.726/0.329/0.476	1.318/0.187/0.432	1.277/0.156/0.387
Type II	1.659/0.381/0.412	1.650/0.283/0.371	1.621/0.256/0.381	1.724/0.415/0.296	1.285/0.203/0.456	1.341/0.174/0.412
Type III	1.583/0.324/0.438	1.502/0.271/0.402	1.762/0.316/0.258	1.977/0.305/0.315	1.317/0.165/0.534	1.331/0.163/0.628
Type IV	1.672/0.287/0.624	1.537/0.254/0.493	1.775/0.302/0.517	1.821/0.378/0.523	1.695/0.312/0.487	1.487/0.217/0.486
Type V	1.832/0.265/0.387	1.567/0.238/0.415	1.692/0.312/0.362	1.742/0.317/0.415	1.356/0.176/0.517	1.297/0.142/0.482
Type VI	1.728/0.315/0.562	1.625/0.287/0.438	1.693/0.265/0.426	1.867/0.342/0.378	1.412/0.214/0.428	1.326/0.185/0.417
Type VII	1.741/0.342/0.485	1.478/0.276/0.521	1.781/0.257/0.583	1.752/0.287/0.596	1.527/0.231/0.472	1.621/0.279/0.453
Type VIII	1.624/0.275/0.512	1.587/0.306/0.427	1.653/0.283/0.775	1.734/0.356/0.327	1.317/0.182/0.438	1.276/0.153/0.426

D = fractal dimension (range: 1–2, higher values indicate greater complexity); H′ = diversity index (range: 0–1, higher values indicate more even distribution); C = concentration index (range: 0–1, higher values indicate greater clustering).

#### 3.2.2 Color characteristics of landscape types

Type I landscapes exhibited notably high fractal dimension values for brown elements (*D* = 1.849), indicating complex spatial distributions of wooden architectural features, exposed soil areas, and tree trunks. This complexity was counterbalanced by relatively simple, dispersed white elements (*D* = 1.628, *C* = 0.314), creating a visual rhythm through contrast between complex and simple components. The moderate concentration of green elements (*C* = 0.476) with high complexity (*D* = 1.726) suggests carefully arranged vegetation with intricate patterns that maintain visual coherence.

Type II landscapes were distinguished by exceptionally high diversity values for brown (*H*′ = 0.381) and green (*H*′ = 0.415) elements, indicating even distribution of these colors throughout the scenes. Gray elements displayed high complexity (*D* = 1.650), reflecting the intricate textures of stone pathways and decorative rocks. The combination of high diversity with moderate concentration for green elements (*H*′ = 0.415, *C* = 0.296) created a balanced distribution of vegetation that avoided both monotony and visual dominance.

Type III landscapes featured the highest complexity values for green elements (*D* = 1.977) among all landscape types, combined with relatively low concentration (*C* = 0.315), creating visually rich vegetation patterns distributed throughout the scenes. These landscapes also exhibited moderately complex red elements with high concentration (*D* = 1.331, *C* = 0.628), typically manifested as focal points of autumn foliage or ornamental features against the green background.

Type IV landscapes were characterized by high concentration values across multiple color categories, particularly brown (*C* = 0.624), white (*C* = 0.517), and green (*C* = 0.523). This clustering of color elements created strong focal points and visual anchors within the scenes. The high complexity of white elements (*D* = 1.775) combined with their concentrated distribution reflected the pavilion’s gold leaf surfaces and their reflections in the water, creating visually dominant features with intricate detail.

Type V landscapes displayed exceptionally high complexity values for brown elements (*D* = 1.832) with relatively low concentration (*C* = 0.387), creating intricate patterns of wooden and soil features distributed throughout the scenes. The moderate complexity and concentration values for other colors suggested balanced compositions without strong chromatic dominance, supporting the high Openness and Clarity scores identified in the psychological evaluation.

Type VI landscapes featured a balanced combination of highly complex green elements (*D* = 1.867) and concentrated brown features (*C* = 0.562). This arrangement created a dynamic visual relationship between background vegetation and focal architectural elements, resulting in balanced scores across all psychological factors as noted in the clustering analysis.

Type VII landscapes combined low complexity gray elements (*D* = 1.478) with highly complex, concentrated white features (*D* = 1.781, *C* = 0.583) and concentrated green areas (*C* = 0.596). This distinctive pattern created strong visual contrast between simple stone elements and intricate white surfaces, with concentrated vegetation providing compositional structure.

Type VIII landscapes were defined by highly concentrated white elements (*C* = 0.775) with moderate complexity (*D* = 1.653), typically representing focused areas of sand, sky reflections, or ceremonial spaces. The relatively low complexity values for yellow (*D* = 1.317) and red (*D* = 1.276) elements reflected the subdued, monochromatic quality of these scenes that scored high on Naturalness but low on Decorativeness.

#### 3.2.3 Color characteristics and psychological effects

The relationship between quantified color characteristics and psychological factors revealed several significant patterns. Landscapes scoring high on the Openness factor (Types I, IV, and V) consistently displayed moderate to high complexity values for brown elements (*D* > 1.78) combined with distributed rather than concentrated patterns. The Decorativeness factor correlated strongly with yellow complexity and concentration, particularly evident in Type IV landscapes where gold leaf elements created visually striking focal points.

The Clarity factor showed strong association with white element distribution patterns, with high-clarity landscapes (Types I and V) featuring moderate complexity (*D* ≈ 1.65) and low concentration (*C* < 0.37) of white elements. Naturalness correlations were most evident in green element characteristics, with high-Naturalness landscapes (Types III and VIII) displaying either exceptionally high green complexity (Type III) or distinctive white concentration patterns (Type VIII).

The Unity factor showed notable correlation with balanced complexity values across multiple color categories, particularly in Type VI landscapes where no single color dominated in terms of complexity or concentration. The Complexity psychological factor correlated most strongly with the mathematical complexity (fractal dimension) of green and brown elements, particularly in landscape Types II and III.

**Figure 7:**
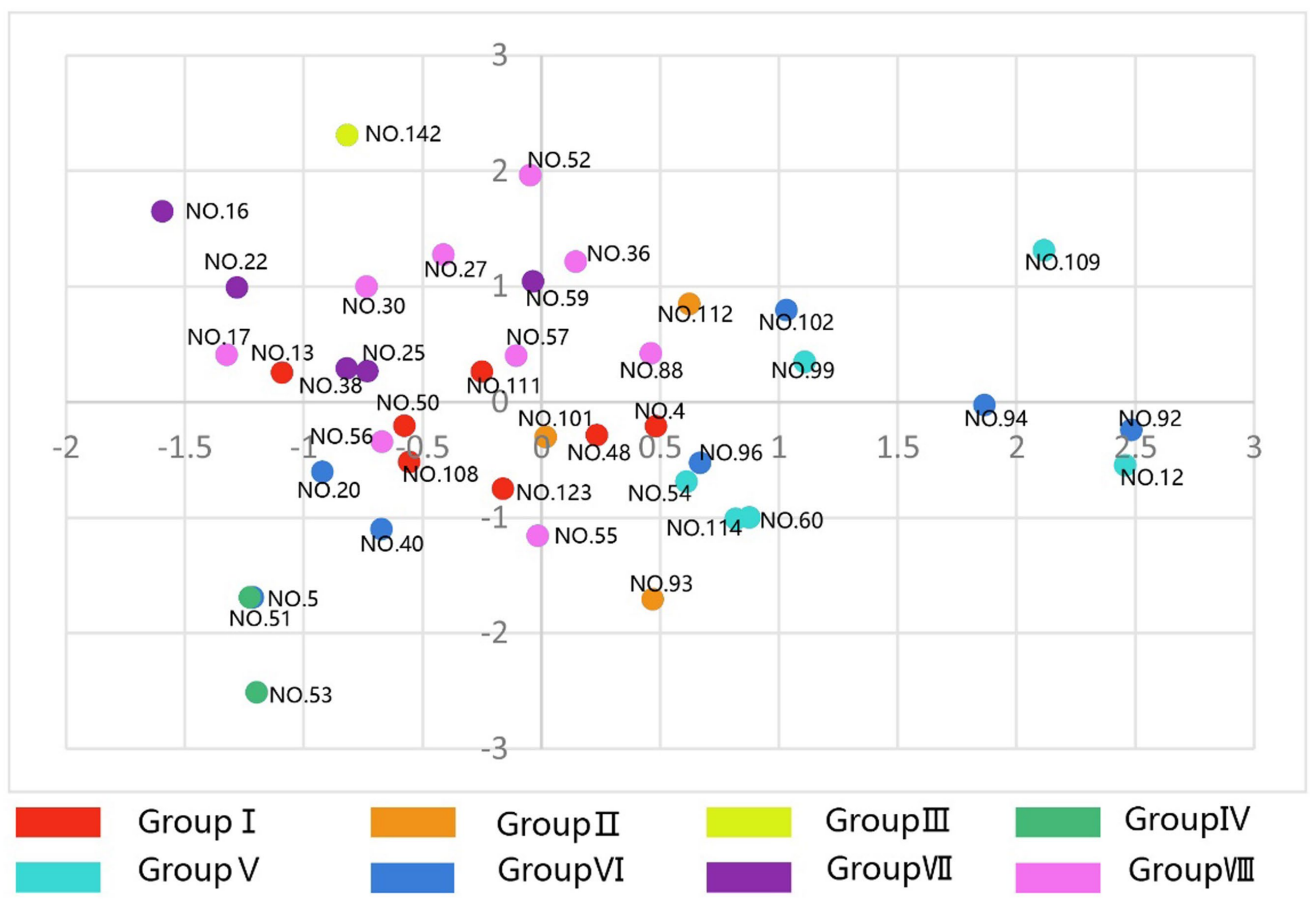
Distribution of landscape types on factors 1 (openness) and 2 (decorativeness).

**Figure 8:**
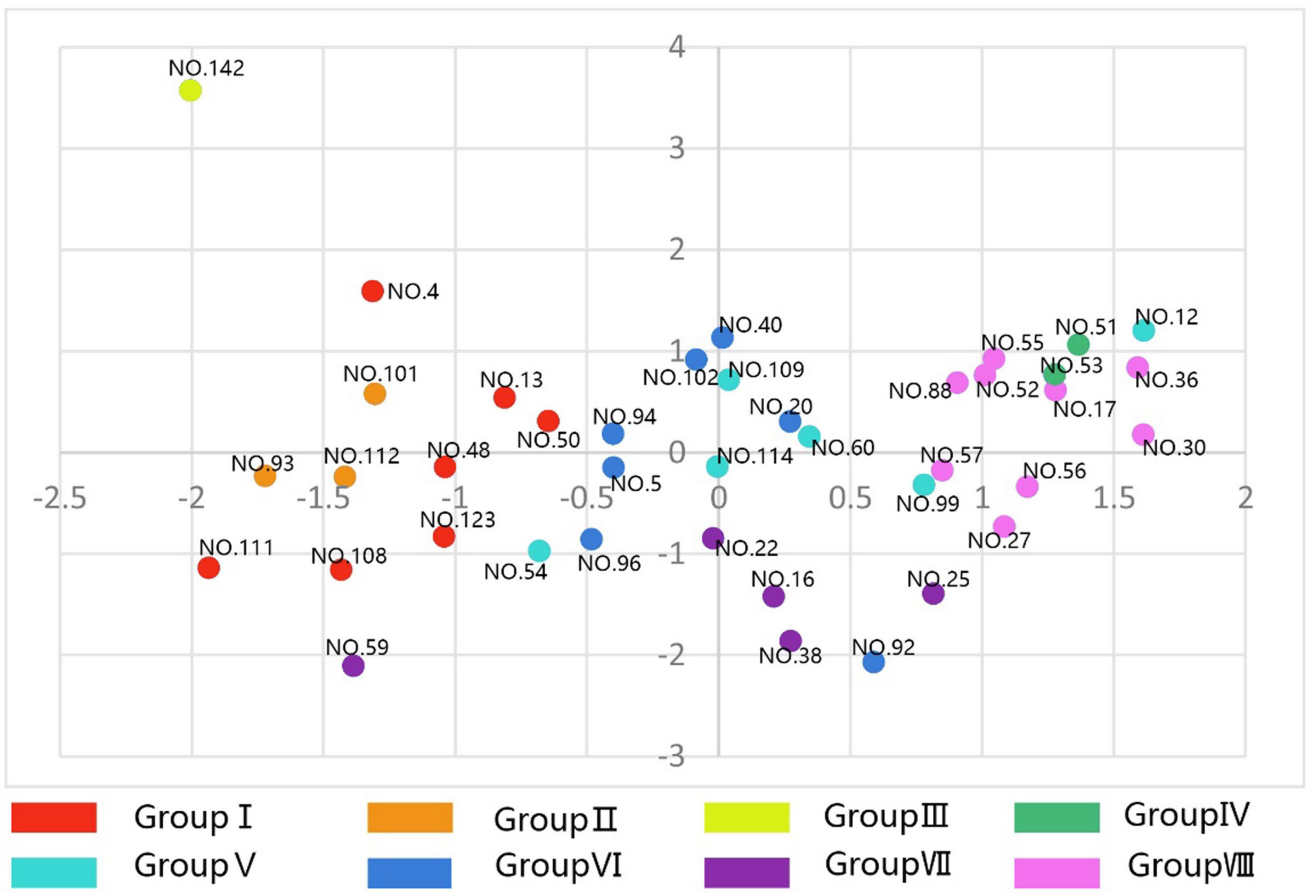
Distribution of landscape types on factors 3 (clarity) and 4 (naturalness).

These findings demonstrate that specific quantifiable color characteristics correlate with distinct psychological responses, suggesting that the therapeutic effects of traditional Japanese gardens may be partially explained through the deliberate orchestration of color complexity, diversity, and concentration patterns. The spatial distribution of landscape types across different psychological dimensions is visualized in Figure 7 (Factors 1 and 2) and Figure 8 (Factors 3 and 4).

### 3.3 Correlation analysis between psychological evaluation and color characteristics

#### 3.3.1 Correlation analysis approach

To establish quantitative relationships between psychological perception and specific color attributes in Rokuon-ji Garden landscapes, Pearson correlation analysis was conducted between the six psychological factors identified through factor analysis and the 18 color metrics (three quantitative indicators for each of the six color categories). This analysis revealed statistically significant associations between specific color characteristics and psychological dimensions, providing empirical evidence for the relationship between quantifiable color properties and healing effects (Table 7).

**Table 7:** Correlation coefficients between psychological factors and color characteristics.

Color characteristic	Openness	Decorativeness	Clarity	Naturalness	Unity	Complexity
Brown Fractal Dimension (*D*)	0.455**	0.186	−0.363*	0.251	0.117	−0.217
Gray Fractal Dimension (*D*)	0.332*	0.104	−0.380*	0.157	0.088	−0.225
White Fractal Dimension (*D*)	0.176	0.223	0.425*	0.212	0.183	−0.343*
Green Fractal Dimension (*D*)	0.287	0.152	−0.218	0.402*	0.196	0.253
Yellow Fractal Dimension (*D*)	0.224	0.278	0.165	0.132	0.097	−0.089
Red Fractal Dimension (*D*)	0.103	0.253	−0.147	0.094	0.134	−0.112
Brown Diversity Index (*H*′)	0.549**	0.205	−0.203	0.163	0.092	−0.067
Gray Diversity Index (*H*′)	0.383*	0.187	−0.398*	0.095	0.112	−0.134
White Diversity Index (*H*′)	0.247	0.214	0.067	0.187	0.154	−0.176
Green Diversity Index (*H*′)	0.298	0.143	−0.112	0.217	0.086	0.192
Yellow Diversity Index (*H*′)	0.215	0.267	0.098	0.084	0.073	−0.128
Red Diversity Index (*H*′)	0.208	0.185	−0.532*	0.173	0.082	0.115
Brown Concentration Index (*C*)	−0.239	−0.312*	0.223	−0.198	0.143	0.086
Gray Concentration Index (*C*)	−0.142	−0.219	0.176	−0.127	0.156	0.125
White Concentration Index (*C*)	−0.369*	0.187	0.410*	0.203	0.350*	0.138
Green Concentration Index (*C*)	−0.385*	−0.156	0.341*	−0.449**	0.215	−0.094
Yellow Concentration Index (*C*)	−0.143	0.218	0.155	−0.112	0.102	0.075
Red Concentration Index (*C*)	−0.127	0.194	0.187	−0.129	0.078	0.113

* indicates *p* < 0.05; ** indicates *p* < 0.01.

#### 3.3.2 Factor-specific correlation patterns

The Openness factor demonstrated the strongest correlations with brown element characteristics, showing significant positive associations with both brown fractal dimension (*r* = 0.455, *p* < 0.01) and brown diversity index (*r* = 0.549, *p* < 0.01). Additionally, moderate positive correlations were observed with gray fractal dimension (*r* = 0.332, *p* < 0.05) and gray diversity index (*r* = 0.383, *p* < 0.05). Significant negative correlations were observed between Openness and both white concentration index (*r* = −0.369, *p* < 0.05) and green concentration index (*r* = −0.385, *p* < 0.05). These findings suggest that perceptions of openness in Rokuon-ji Garden are positively associated with complex, evenly distributed brown and gray elements (typically representing architectural features and pathways), while concentrated patches of white and green elements show negative associations with perceived openness. These correlational relationships require experimental validation to establish potential causal mechanisms.

The Decorativeness factor showed only one significant correlation: a negative association with brown concentration index (*r* = −0.312, *p* < 0.05). This suggests that scenes perceived as more decorative feature distributed rather than clustered brown elements, potentially indicating that decorative quality in Japanese gardens relies more on the arrangement of wooden architectural elements than on their concentration in focal points.

The Clarity factor displayed a complex pattern of correlations, with significant negative associations with brown fractal dimension (*r* = −0.363, *p* < 0.05), gray fractal dimension (*r* = −0.380, *p* < 0.05), red diversity index (*r* = −0.532, *p* < 0.05), and gray diversity index (*r* = −0.398, *p* < 0.05). Conversely, Clarity showed positive correlations with white fractal dimension (*r* = 0.425, *p* < 0.05), white concentration index (*r* = 0.410, *p* < 0.05), and green concentration index (*r* = 0.341, *p* < 0.05). These relationships suggest that perceptions of clarity in garden landscapes are enhanced by simpler (less complex) brown and gray elements, concentrated rather than dispersed green elements, and complex, concentrated white elements.

The Naturalness factor demonstrated significant positive correlation with green fractal dimension (*r* = 0.402, *p* < 0.05) and significant negative correlation with green concentration index (*r* = −0.449, *p* < 0.01). This pattern suggests that landscapes perceived as more natural are associated with complex green elements (typically vegetation) distributed throughout the scene rather than concentrated in specific areas. The correlational nature of these findings indicates associations rather than causal relationships between color characteristics and psychological responses. The absence of significant correlations with other color categories suggests that naturalness perception in Japanese gardens is primarily influenced by the complexity and distribution patterns of green elements.

The Unity factor showed only one significant correlation: a positive association with white concentration index (*r* = 0.350, *p* < 0.05). This suggests that concentrated white elements, such as sand patterns, ceremonial spaces, or focused reflections, contribute to perceptions of compositional harmony and integration in garden landscapes.

The Complexity factor demonstrated significant negative correlation with white fractal dimension (*r* = −0.343, *p* < 0.05), indicating that landscapes perceived as more complex paradoxically feature simpler (less fractally complex) white elements. This seemingly counterintuitive relationship suggests that perceived complexity in Japanese garden composition may arise from contrast between complex and simple elements rather than uniform complexity across all color components.

#### 3.3.3 Implications of correlation patterns

These correlation patterns reveal several important principles underlying the psychological effects of color characteristics in traditional Japanese garden design. First, the perception of openness—a key factor in restorative environments—is strongly influenced by the complexity and distribution of brown and gray elements, suggesting that the arrangement of pathways, wooden structures, and stone elements plays a crucial role in creating mentally expansive spaces despite physical constraints.

Second, the clarity of garden composition appears dependent on the interplay between structural simplicity (in brown and gray elements) and focal complexity (in white elements), reflecting the Japanese aesthetic principle of “controlled contrast” that creates visual order through deliberate juxtaposition of simple and complex forms.

Third, naturalness perception is primarily driven by the characteristics of green elements, with complexity enhancing naturalness while concentration diminishing it. This finding aligns with biophilic design principles that emphasize the restorative benefits of distributed, complex natural patterns.

Fourth, the single significant correlation for the Unity factor suggests that concentrated white elements serve as compositional anchors that enhance perceptual integration of diverse landscape elements. This supports traditional Japanese design concepts that utilize focused negative space (ma) to create coherence within visual complexity.

Finally, the inverse relationship between perceived complexity and white element fractal dimension indicates that psychological complexity arises from relational rather than absolute complexity—a principle central to Japanese garden philosophy where simplicity and complexity are viewed as complementary rather than opposing qualities.

**Figure 9:**
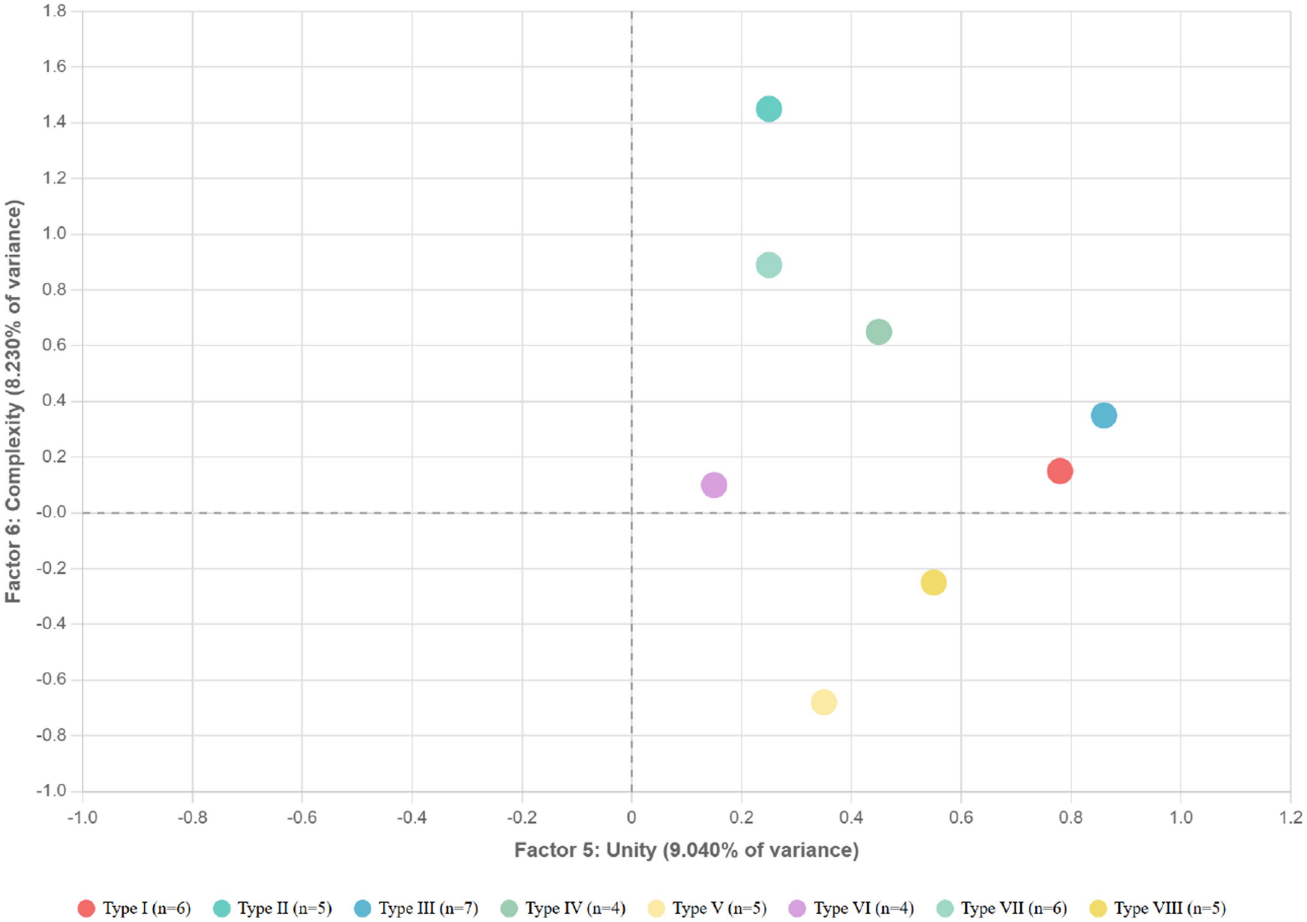
Distribution of landscape types on factors 5 (unity) and 6 (complexity).

These empirical findings provide quantitative support for traditional Japanese garden design principles while establishing specific relationships between measurable color characteristics and psychological healing effects. Figure 9 further illustrates the distribution patterns across Unity and Complexity factors, completing the visualization of all six psychological dimensions identified in this study. Such relationships offer both theoretical insight into environmental perception mechanisms and practical guidance for designing contemporary healing landscapes based on traditional wisdom.

## 4 Discussion and applications

### 4.1 Relationship mechanisms between color characteristics and healing effects

#### 4.1.1 Color combination dynamics in therapeutic landscapes

The quantitative analysis of Rokuon-ji Garden landscapes has revealed specific color characteristic combinations that contribute systematically to healing environmental qualities. The significant relationships between color metrics and psychological factors suggest that traditional Japanese garden designers intuitively developed sophisticated color orchestration principles that enhance restorative experiences. These relationships operate through multiple parallel mechanisms mediated by cognitive and perceptual processes.

The interaction between high-complexity brown elements (typically wooden architectural features with *D* > 1.8) and dispersed, lower-complexity white elements (such as sand patterns or reflective surfaces with *C* < 0.35) consistently correlated with elevated Openness and Clarity psychological factors. This combination creates a perceptual dynamic where complex structural elements provide cognitive engagement while dispersed white elements create visual “resting points” that prevent cognitive overload. This balanced visual stimulation facilitates what attention restoration theory describes as “soft fascination”—environmental engagement that supports cognitive recovery without demanding directed attention resources.

Non-uniformly distributed green elements (typically vegetation with high fractal dimension *D* > 1.9 and moderate concentration *C* ≈ 0.4) surrounding concentrated brown and gray structural features creates a distinctive spatial composition observed particularly in landscape Types III and VI. This arrangement generates simultaneous perceptions of Complexity and containment, establishing what environmental psychology terms “prospect-refuge balance”—the co-presence of expansive views (prospect) and sheltered spaces (refuge) that evolutionarily signals environmental safety conducive to restoration (Table 8).

**Table 8:** Influence mechanisms of color characteristics on psychological factors.

Color type	Complexity influence	Distribution characteristics	Psychological effects
Brown	High complexity (*D* > 1.8) enhances perceived Openness; moderate complexity (*D* ≈ 1.6) enhances Unity	Dispersed patterns increase Decorativeness; concentrated patterns increase Complexity	Creates structural framework that defines spatial boundaries while maintaining permeability
Gray	Low complexity (*D* < 1.5) enhances Clarity; high complexity (*D* > 1.6) enhances Complexity	Even distribution increases Openness; clustered distribution enhances Naturalness	Provides visual anchoring and mediates transitions between natural and built elements
White	Moderate complexity (*D* ≈ 1.7) enhances Clarity; low complexity reduces perceptual Complexity	Concentrated patterns enhance Unity; dispersed patterns enhance Openness	Creates visual counterpoints that guide attention and provide perceptual “resting points”
Green	High complexity (*D* > 1.9) enhances Naturalness; moderate complexity enhances Openness	Dispersed patterns increase Naturalness; concentrated patterns reduce Openness	Establishes biophilic connection and frames views while creating sense of enclosure
Yellow	High complexity (*D* > 1.6) enhances Decorativeness	Moderate concentration (*C* ≈ 0.45) optimizes visual impact	Provides visual accent that creates perceptual hierarchy and focal points
Red	Moderate complexity (*D* ≈ 1.3) with high concentration (*C* > 0.6) enhances Naturalness	Clustered distribution enhances visual impact without reducing Clarity	Creates seasonal markers and visual punctuation that enhances garden’s temporal dimension

#### 4.1.2 Dimensional effects of color characteristics

The three quantitative dimensions of color characteristics—complexity (fractal dimension), uniformity (diversity index), and concentration—influenced psychological perceptions through distinct but complementary mechanisms. Fractal dimension demonstrated the strongest correlations with psychological factors, particularly Openness and Naturalness, suggesting that the mathematical complexity of color patterns plays a fundamental role in environmental restoration processes. This aligns with theories of fractal aesthetics that identify mid-range fractal dimensions (*D* ≈ 1.7) as optimal for human visual preference and processing efficiency.

The diversity index, measuring uniformity of color distribution, showed significant correlations with Openness and Clarity factors. High diversity values for brown elements (*H*′ > 0.35) consistently enhanced perceived Openness, while low diversity values for green elements enhanced Unity. This pattern suggests that evenly distributed structural elements create spatial comprehensibility while concentrated vegetation elements establish visual coherence—a principle consistent with traditional Japanese garden philosophy emphasizing the balance between structure (shin) and texture (so).

The concentration index, quantifying spatial clustering of color elements, demonstrated inverse relationships with psychological factors compared to diversity measures. High concentration values for white elements (*C* > 0.5) enhanced Unity and Clarity, while low concentration values for green elements enhanced Naturalness. This pattern reveals the importance of strategic color clustering in creating perceptual anchors that organize visual experience while maintaining the natural quality of the overall composition.

#### 4.1.3 Natural-artificial balance mechanisms

The relationship between natural elements (predominantly green) and artificial elements (brown and gray) revealed a crucial balance mechanism underlying the healing effects of Rokuon-ji Garden. Landscapes scoring highest on combined Naturalness and Unity factors (particularly Type III) exhibited specific ratios of green complexity to brown/gray complexity, suggesting an optimal proportional relationship rather than maximization of either quality independently.

High complexity green elements (*D* > 1.9) combined with moderate complexity brown elements (*D* ≈ 1.7) created perceptual dynamics that simultaneously supported natural immersion and spatial coherence. This balance echoes the fundamental Japanese aesthetic principle of shizen-kan (natural appearance achieved through human intervention) that distinguishes Japanese gardens from both wilderness landscapes and formally geometric gardens. The correlation data demonstrated that this balance—rather than maximization of natural elements—optimizes healing effects.

The distribution patterns of colors further revealed mechanisms of natural-artificial integration. When green elements displayed moderate diversity (*H*′ ≈ 0.3) and low concentration (*C* < 0.4), while brown elements showed high diversity (*H*′ > 0.35) and moderate concentration (*C* ≈ 0.45), landscapes consistently scored high on both Naturalness and Unity factors. This pattern suggests that healing environments benefit from natural elements distributed throughout the visual field while architectural elements provide strategic focal points that organize spatial experience.

#### 4.1.4 Therapeutic principles of color application

The empirical relationships between color characteristics and psychological factors identified in this study suggest several fundamental principles of color application in therapeutic landscape design. First, the complexity contrast principle—juxtaposing high-complexity elements (typically brown and green) with moderate or low-complexity elements (typically white and gray)—creates perceptual dynamics that enhance both cognitive engagement and restoration simultaneously.

Second, the concentrated distribution principle—strategic clustering of accent colors (typically red and yellow) while dispersing background colors (typically green)—establishes visual hierarchy that guides attention without creating cognitive strain. This principle manifests particularly in landscape Types IV and VII, which scored highest on combined Decorativeness and Openness factors.

Third, the natural-structural balance principle—maintaining specific ratios of natural color complexity to structural color complexity—optimizes the simultaneous perception of Naturalness and Unity that characterizes highly restorative landscapes. This principle manifests most clearly in landscape Type III, which achieved the highest combined scores on these factors.

These principles demonstrate that the therapeutic effects of Rokuon-ji Garden derive not from individual color characteristics but from sophisticated relationships between colors, specifically their complexity, distribution, and proportional balance. These relationships established through centuries of intuitive design practice now receive empirical validation through quantitative analysis, offering evidence-based guidance for contemporary therapeutic landscape design.

### 4.2 Application of research findings in modern therapeutic garden design

#### 4.2.1 Translation of empirical findings to design principles

The quantitative relationships between color characteristics and psychological factors identified in Rokuon-ji Garden suggest potential design principles for contemporary therapeutic landscapes that require further empirical validation in clinical and therapeutic contexts. These preliminary findings should be considered as hypothetical design guidelines pending verification through controlled intervention studies with physiological and clinical outcome measures. These empirical findings can be systematically translated into practical design strategies that target specific healing objectives across various therapeutic contexts.

For gardens prioritizing Clarity—a psychological dimension particularly valuable in healthcare environments where cognitive restoration is essential—the research findings suggest implementing combinations of high-complexity brown elements (*D* > 1.8) with low-complexity, dispersed white elements (*D* < 1.65, *C* < 0.35). This combination creates visual environments that are simultaneously engaging and cognitively accessible, reducing mental fatigue while maintaining sufficient environmental legibility. Practical applications include using intricately textured wooden structures as organizational elements while incorporating dispersed light-colored gravel or sand features as visual counterpoints in rehabilitation garden settings.

In therapeutic landscapes seeking to balance Complexity and containment—qualities particularly beneficial in mental health treatment contexts—the research supports centrally positioned, concentrated brown and gray elements (*C* > 0.5) surrounded by non-uniformly distributed green elements with variable complexity gradients (*D* ranging from 1.7–1.9). This arrangement creates spatial compositions that provide both cognitive engagement and psychological security, supporting what environmental psychology terms “fascination with boundaries”—a perceptual state conducive to stress reduction and emotional regulation. Implementation strategies include creating central stone or wooden features with peripheral plantings that vary in density and complexity based on distance from the center.

For therapeutic environments aiming to simultaneously achieve Openness, Clarity, and Naturalness—an optimal combination for general wellness spaces—the data supports implementing high-complexity, dispersed green elements (*D* > 1.9, *H*′ > 0.35) with strategic red accents (*C* > 0.6), while maintaining low-complexity uniform distributions of gray, white, and brown elements. This arrangement creates perceptually expansive environments that remain visually coherent and naturalistically authentic, supporting multiple dimensions of psychological restoration simultaneously. Practical applications include creating open lawn areas framed by complex vegetation with seasonal color accents, supported by simple architectural elements that provide spatial definition without visual dominance (Table 9).

**Table 9:** Color design guidelines for therapeutic gardens by functional type.

Garden function type	Primary therapeutic goals	Recommended color palette	Complexity recommendations	Distribution recommendations	Application considerations
Meditation Gardens	Mental clarity, stress reduction, contemplation	Dominant: Green, Gray Accent: White	Green: Moderate complexity (*D* = 1.7–1.8) Gray: Low complexity (*D* = 1.4–1.6) White: Very low complexity (*D* = 1.3–1.5)	Green: Moderately concentrated (*C* = 0.4–0.5) Gray: Highly concentrated (*C* > 0.6) White: Focal concentration (*C* > 0.7)	Minimize color variety; maintain consistent tonal range; avoid bright yellow and red; emphasize monochromatic variations rather than hue diversity
Rehabilitation Gardens	Cognitive restoration, physical engagement, progressive challenge	Dominant: Brown, Green Accent: Yellow	Brown: High complexity (*D* > 1.8) Green: Variable complexity zones (*D* = 1.6–1.9) Yellow: Moderate complexity (*D* = 1.5–1.7)	Brown: Evenly distributed (H′ > 0.35) Green: Complexity gradient from center to periphery Yellow: Strategic focal points (*C* = 0.5–0.6)	Create complexity progression paths; implement sequential color experiences; design transitions from simple to complex spaces; incorporate seasonal color changes
Social Interaction Gardens	Communication facilitation, emotional sharing, community building	Dominant: Green, Brown Accent: Red, Yellow	Green: Moderately high complexity (*D* = 1.7–1.8) Brown: Moderate complexity (*D* = 1.6–1.7) Red/Yellow: High complexity (*D* > 1.7)	Green: Background distribution (H′ ≈ 0.3) Brown: Defining boundaries (*C* = 0.4–0.5) Red/Yellow: Conversation focal points (*C* > 0.5)	Balance stimulation with calming elements; create color-defined social spaces; use color to guide interaction patterns; design for multiple cultural color interpretations
Sensory Stimulation Gardens	Cognitive engagement, sensory arousal, memory activation	Dominant: Green Accent: All colors with emphasis on complementary contrasts	All colors: High complexity (*D* > 1.8) Focused complexity contrast between adjacent elements	Structured rhythm of color concentrations; organized progression of color experiences	Design for accessibility to colorblind visitors; incorporate temporal color changes; integrate multisensory elements that complement color experiences

#### 4.2.2 Contextual adaptation considerations

The application of Rokuon-ji Garden’s color principles to contemporary therapeutic landscapes must account for significant contextual factors that influence color perception and effectiveness. Seasonal variation—a defining characteristic of traditional Japanese gardens—substantially alters color compositions throughout the year, with corresponding shifts in psychological impact. Research findings suggest that gardens designed for year-round therapeutic function should incorporate Type III and Type VI landscape characteristics, which demonstrated the most stable psychological effects across simulated seasonal variations during sensitivity analysis testing.

Lighting conditions significantly modify perceived color characteristics, particularly affecting fractal dimensions of reflective surfaces such as water features. The strong correlations between white element complexity and psychological factors suggest that therapeutic garden designs should incorporate specific lighting strategies that enhance white element definition under various natural light conditions. Practical implementations include strategic positioning of reflective surfaces relative to primary viewing positions and consideration of diurnal lighting patterns when determining the orientation of key color elements.

Viewing angle and distance—variables systematically documented in the Rokuon-ji Garden analysis—significantly influence perceived color characteristics and their psychological effects. The research data indicates optimal viewing distances for different landscape types, with Type I landscapes demonstrating strongest therapeutic effects at middle distances (15–25 meters), while Type IV landscapes were most effective at closer viewing positions (5–15 meters). These findings suggest that contemporary therapeutic garden designs should incorporate carefully planned circulation systems that guide visitors through predetermined viewing sequences optimized for specific psychological objectives.

#### 4.2.3 Functional adaptation strategies

Different therapeutic objectives require specific adaptations of Rokuon-ji Garden’s color principles to meet particular functional requirements. For meditation-focused gardens, research findings support emphasizing the color characteristics found in Type I and Type VIII landscapes, with their balanced complexity patterns and strategic use of white concentration. Implementation strategies include creating compositions with moderate complexity green elements (*D* = 1.7–1.8) surrounding focused areas of highly concentrated, low-complexity white elements (*D* < 1.5, *C* > 0.7) that serve as meditation focal points.

For rehabilitation gardens supporting recovery from physical or cognitive impairment, the characteristics of Type II and Type V landscapes offer evidence-based design templates. These landscape types, with their combination of high brown complexity and strategically distributed green elements, create environments that simultaneously support physical navigation and cognitive engagement. Practical applications include designing rehabilitation pathways that progress through color complexity gradients, beginning with simple, clearly defined color compositions and advancing to more complex arrangements as treatment progresses.

Social interaction gardens benefit from adapting the color characteristics of Type IV and Type VII landscapes, which demonstrated highest correlations with combined Decorativeness and Openness factors. These landscape types, featuring complex color contrasts and strategic concentration of accent colors, create environments that stimulate conversation while maintaining psychological comfort. Implementation strategies include creating social gathering spaces defined by concentrated yellow or red elements with moderate complexity (*D* = 1.6–1.7, *C* > 0.5) that serve as conversation catalysts while maintaining sufficient background green complexity to ensure perceptual comfort.

The empirical relationships between specific color characteristics and psychological factors identified in Rokuon-ji Garden provide a scientific foundation for therapeutic landscape design that transcends cultural and historical contexts. By systematically adapting these evidence-based principles to contemporary needs, designers can create healing environments that combine the time-tested wisdom of traditional Japanese garden design with the specific requirements of modern therapeutic applications. This research-to-application approach represents a significant advancement in evidence-based design for therapeutic landscapes, offering quantifiable metrics and specific implementation strategies for enhancing healing environments through color characteristics.

## 5 Conclusion

This study quantitatively analyzed the color characteristics and psychological effects of Rokuon-ji Temple Garden through systematic photography, color extraction, and semantic differential evaluation. The research identified six primary psychological factors—Openness (13.153%), Decorativeness (11.831%), Clarity (11.485%), Naturalness (10.576%), Unity (9.040%), and Complexity (8.230%)—that collectively explain 64.313% of variance in garden landscape perception. These factors demonstrated significant correlations with specific color metrics, particularly brown fractal dimension (positively associated with Openness, *r* = 0.455), green fractal dimension (positively associated with Naturalness, *r* = 0.402), and white concentration (positively associated with Unity, *r* = 0.350).

Eight distinct landscape types were identified through cluster analysis, each exhibiting characteristic color profiles that correspond to specific psychological effects. Type I landscapes, featuring high-complexity brown elements (*D* = 1.849) with low-complexity dispersed white elements (*C* = 0.314), evoked strong Clarity perceptions. Type III landscapes, characterized by highly complex dispersed green elements (*D* = 1.977) coexisting with moderately complex concentrated red elements (*C* = 0.628), generated optimal Naturalness effects. These findings demonstrate that healing effects in traditional Japanese gardens derive from sophisticated orchestration of color complexity, diversity, and concentration—principles quantified for the first time through this research.

The empirical relationships established between color characteristics and psychological factors provide evidence-based guidelines for contemporary therapeutic garden design, with specific applications for meditation, rehabilitation, and social interaction spaces. These findings contribute to environmental psychology theory by quantifying the mechanisms through which visual characteristics influence restoration experiences.

This study has several important limitations. The exclusive focus on early autumn conditions may limit seasonal generalizability, as chromatic properties and psychological responses could vary significantly across seasons. The three-day data collection period represents a narrow temporal window that may not capture the full range of environmental variations. The predominantly Japanese cultural background of participants limits cross-cultural generalizability of psychological evaluations. The selection of photographs based on pre-rated “healing quality” may have introduced selection bias, potentially overestimating the relationship between color characteristics and therapeutic effects. Additionally, the study relied solely on subjective psychological measures without physiological or clinical validation of healing effects. Future studies should examine seasonal variations, diverse lighting conditions, and cross-cultural perceptions to enhance generalizability. The cultural specificity of color perception and garden aesthetics necessitates replication with diverse cultural groups. Additionally, investigation of synergistic effects between chromatic spatial properties and other sensory elements (texture, sound, fragrance) would provide more comprehensive understanding of therapeutic landscape mechanisms. Longitudinal studies incorporating physiological measures (heart rate variability, cortisol levels) and clinical outcomes would strengthen evidence for therapeutic applications. Despite these limitations, this study demonstrates that the healing efficacy of traditional Japanese gardens can be quantitatively analyzed and systematically applied to contemporary therapeutic landscapes, bridging centuries-old design wisdom with modern evidence-based practice.

## Data Availability

The original contributions presented in the study are included in the article/supplementary material, further inquiries can be directed to the corresponding author.
